# Identification of a Chrysanthemic Ester as an Apolipoprotein E Inducer in Astrocytes

**DOI:** 10.1371/journal.pone.0162384

**Published:** 2016-09-06

**Authors:** Jianjia Fan, Shahab Zareyan, Wenchen Zhao, Yoko Shimizu, Tom A. Pfeifer, Jun-Hyung Tak, Murray B. Isman, Bernard Van den Hoven, Mark E. Duggan, Michael W. Wood, Cheryl L. Wellington, Iva Kulic

**Affiliations:** 1 Department of Pathology and Laboratory Medicine, University of British Columbia, Djavad Mowafaghian Centre for Brain Health, Vancouver, British Columbia, Canada; 2 Centre for Drug Research and Development, Vancouver, British Columbia, Canada; 3 Faculty of Land and Food Systems, University of British Columbia, Vancouver, British Columbia, Canada; 4 TC Scientific Inc., Edmonton, Alberta, Canada; 5 AstraZeneca, Cambridge, Massachusetts, United States of America; Sungkyunkwan University, REPUBLIC OF KOREA

## Abstract

The apolipoprotein E (*APOE*) gene is the most highly associated susceptibility locus for late onset Alzheimer’s Disease (AD), and augmenting the beneficial physiological functions of apoE is a proposed therapeutic strategy. In a high throughput phenotypic screen for small molecules that enhance apoE secretion from human CCF-STTG1 astrocytoma cells, we show the chrysanthemic ester 82879 robustly increases expressed apoE up to 9.4-fold and secreted apoE up to 6-fold and is associated with increased total cholesterol in conditioned media. Compound 82879 is unique as structural analogues, including pyrethroid esters, show no effect on apoE expression or secretion. 82879 also stimulates liver x receptor (LXR) target genes including ATP binding cassette A1 (ABCA1), LXRα and inducible degrader of low density lipoprotein receptor (IDOL) at both mRNA and protein levels. In particular, the lipid transporter ABCA1 was increased by up to 10.6-fold upon 82879 treatment. The findings from CCF-STTG1 cells were confirmed in primary human astrocytes from three donors, where increased apoE and ABCA1 was observed along with elevated secretion of high-density lipoprotein (HDL)-like apoE particles. Nuclear receptor transactivation assays revealed modest direct LXR agonism by compound 82879, yet 10 μM of 82879 significantly upregulated apoE mRNA in mouse embryonic fibroblasts (MEFs) depleted of both LXRα and LXRβ, demonstrating that 82879 can also induce apoE expression independent of LXR transactivation. By contrast, deletion of LXRs in MEFs completely blocked mRNA changes in ABCA1 even at 10 μM of 82879, indicating the ability of 82879 to stimulate ABCA1 expression is entirely dependent on LXR transactivation. Taken together, compound 82879 is a novel chrysanthemic ester capable of modulating apoE secretion as well as apoE-associated lipid metabolic pathways in astrocytes, which is structurally and mechanistically distinct from known LXR agonists.

## Introduction

Alzheimer’s disease (AD) is the most common form of dementia and results in severe impairments in memory [1]. The diagnosis of AD is confirmed by postmortem histological identification of two neuropathological hallmarks: 1) extracellular amyloid plaques composed mainly of aggregated β-amyloid (Aβ) peptides; and 2) intracellular neurofibrillary tangles consisting of hyperphosphorylated tau [2]. Despite considerable evidence supporting the Aβ pathway as a therapeutic target, all clinical trials targeting Aβ have failed, underscoring the paramount importance of evaluating alternative targets [3, 4].

Apolipoprotein E (apoE) plays a significant role in AD pathogenesis and is a promising candidate therapeutic target. ApoE is the most abundant apolipoprotein expressed in the central nervous system (CNS) [5, 6], where it is predominantly synthesized and secreted by astrocytes [7]. Following local production in the CNS, apoE is lipidated by the ATP-binding cassette transporter A1 (ABCA1) to form lipoprotein particles that resemble high-density lipoproteins (HDL) in size and buoyant density [8]. These particles transfer their lipid cargo to target cells via apoE receptors belonging to the low-density lipoprotein receptor (LDLR) family [9, 10].

*APOE* is also the primary genetic risk factor for the vast majority of AD cases in the population, which are sporadic and termed late onset AD [11]. Three allelic isoforms of *APOE* are found in the human population: *APOE2* (cys112, cys158), *APOE3* (cys112, arg158), and *APOE4* (arg112, arg158) [12]. *APOE4* is associated with aberrant cholesterol homeostasis, enhanced amyloid deposition and inflammation, as well as impaired blood-brain barrier (BBB) integrity and cerebrovascular dysfunction, reflecting the numerous and only partly understood functions of apoE in the CNS [10, 13–17]. As at least one copy of *APOE4* is present in ~17% of the population and in up to 60% of AD patients [18–20], overcoming the detrimental effects of *APOE4* has become one of the most important challenges in the AD field. Studies in mice and humans suggest that *APOE4* carriers can have lower levels of CNS apoE [21–26], which is presumed to be due to increased degradation of poorly-lipidated apoE in *APOE4* carriers than in *APOE2* or *APOE3* carriers. Decreased cerebrospinal fluid (CSF) apoE levels in *APOE4* carriers are associated with reduced CSF Aβ42, which is thought to reflect its accumulation in amyloid plaques [27, 28]. Indeed, reduced CSF apoE levels predict worse clinical outcome while increased CSF apoE is suggested to be a protective response to injury in AD [27, 28]. Hence, elevating functional apoE levels by increasing its production and lipidation by glial cells may be of therapeutic importance.

ABCA1 is a key regulator of brain apoE levels and lipidation status. Previous studies have shown that *Abca1* deficiency in mice decreases apoE levels by at least 65% in both CSF and brain tissue, which can increase Aβ deposition [29–32]. Further, loss of a single copy of *Abca1* leads to cognitive deficits and increased amyloid burden in *APOE4*- but not in *APOE3*-expressing AD mice [33], suggesting that apoE4 functions may be particularly sensitive to its lipidation status. Additionally, inducing *Abca1* using either pharmacological or genetic methods can improve cognitive function and reduce amyloid levels in AD mouse models [34] and increase apoE lipidation in the brain [35]. Finally, loss-of-function mutations in *ABCA1* are associated with decreased plasma apoE and increased dementia risk [36]. Hence, methods to upregulate ABCA1 are also of interest as a potential therapeutic approach for AD.

ApoE and ABCA1 expression, as well as other lipid metabolic genes, are regulated through nuclear liver x receptors (LXRs), which are activated by endogenous cholesterol-derived oxysterols [37, 38]. While the two isoforms of LXR, LXRα and LXRβ, are both expressed in the brain, LXRα is enriched in the liver whereas LXRβ is ubiquitously expressed [39, 40]. Treatment of AD mice with synthetic LXR agonists, including TO901317 and GW3965, consistently improves cognitive performance and can decrease amyloid burden [41–46], and we have shown that ABCA1 is required for the beneficial effects of GW3965 in AD mice [42]. Conversely, genetic deletion of LXRs exacerbates AD pathogenesis [47].

LXRs function as obligate heterodimers with the retinoid x receptor (RXR). Of note is the RXR agonist bexarotene that has been shown to upregulate ABCA1 and ABCG1 thereby increasing apoE4 lipidation, which reverses apoE4-induced cognitive and neuronal impairments in non-amyloid mice [48], and lowers Aβ oligomers and strengthens synaptic viability in E4FAD AD mice [49]. Recently, in a study using *Abca1* deficient AD mice, bexarotene was unable to induce apoE expression or increase apoE lipidation in the absence of *Abca1* [50]. ABCA1 was also required for the beneficial effects of bexarotene in clearing soluble brain Aβ and improving cognition [50]. Although studies using bexarotene have led to mixed results on amyloid and Aβ levels in AD mice [51–56], the emerging consensus is that increasing apoE lipidation via LXR or RXR agonists provides therapeutic benefits in mice, even in the case of apoE4.

However, despite the benefits of activating the LXR/RXR pathway, hepatotoxic and systemic side effects have hampered clinical development of direct LXR/RXR agonists. The major liability of current direct LXR agonists is the induction of hypertriglyceridemia and liver steatosis due to activation of hepatic sterol regulatory element binding protein-1c (SREBP-1c), an LXR target that, in the liver, induces lipogenesis [57]. Although approved by the FDA for use in cutaneous T-cell lymphoma, bexarotene also has known adverse effects on blood lipid profiles that may pose long-term safety challenges [58]. As therapeutics that increase brain ABCA1 and apoE without triggering undesirable hepatic effects are therefore of great interest, studies that inform upon potential cell-type specific effects of compounds of interest are highly desirable.

We performed a high-throughput screen using a library of 104,000 small molecules to identify compounds that increase apoE secretion from human CCF-STTG1 astrocytoma cells. Upon deconvolution of hits discovered in an orthogonal screening approach, we identified a single chrysanthemic ester, compound 82879, which stimulates apoE secretion as well as both apoE and ABCA1 expression in both CCF-STTG1 cells and primary human astrocytes. Although compound 82879 does show modest direct LXR transactivation, its ability to induce apoE expression includes both LXR-dependent and LXR-independent components. Compound 82879 may therefore be an important research tool to investigate the effects of modulating apoE levels via non-LXR pathways, which may provide insights into developing novel therapeutic approaches for AD targeting apoE that can potentially bypass the undesirable effects of traditional direct LXR agonists.

## Materials and Methods

### Cell Lines and Reagents

Human CCF-STTG1 astrocytoma, human Caco2 epithelial colorectal adenocarcinoma, human HepG2 hepatoma and mouse BV2 microglia cell lines were purchased from ATCC. Immortalized LXR double-knockout (LXRα-/LXRβ-) and LXR expressing (LXRα+/LXRβ-) mouse embryonic fibroblasts (MEFs), previously described [59], were kindly provided by Dr. Peter Tontonoz (UCLA, CA). Primary human astrocytes and primary human hepatocytes (hHepatocyte) were purchased from ScienCell. Primary human macrophages (hMacrophage) were purchased from STEMCELL (BC, Canada). The LXR agonist GW3965 was provided by Dr. Jon Collins (GlaxoSmithKline, NC). The small molecule screening library from which compound 82879 was identified and from which additional structural analogues were tested was provided by LIMR Chemical Genomics Center (LCGC, Wynnewood, PA). Permethrin was purchased from Sigma-Aldrich (cat #: 45614). Bifenethrin (cat #: N-11203) and cyhalothrin (cat #: N-12307) were purchased from Chem Service (West Chester, PA). Tefluthrin was purchased from Santa Cruz (cat #: sc-236965). Stocks of all compounds were prepared in dimethyl sulfoxide (DMSO).

### High Throughput Screen

For the primary screen, CCF-STTG1 cells were seeded into 384-well plates (17,500 cells/well) in growth media (described below) and incubated for 2 h. Compound pools (1 μM final concentration) from an orthogonally pooled small molecule library containing a total of 104,000 compounds [60] from LCGC (http://www.lcgcinc.com/Pages/OPS.html) and the reference compound GW3965 (800 nM final concentration) were added into the growth media and incubated for 96 h at 37°C. Plates were centrifuged at 1,000 *g* for 3 min. Conditioned media was collected and secreted apoE was quantified by ELISA. Compounds that produced at least a two-fold increase in secreted apoE over DMSO controls were individually retested (318 total compounds) using an 8-point concentration response assay performed at half-log intervals (0.1 to 30 μM) under the same conditions as the initial screen.

### Cell Culture and Treatment

CCF-STTG1, MEF and a BV2 murine microglia cell line were cultured in growth media consisting of Dulbecco’s modified Eagle’s medium (DMEM) with 10% fetal bovine serum (FBS, Gibco), 2 mM L-glutamine and 1% penicillin/streptomycin (Invitrogen). HepG2 cells were maintained in the same growth media supplemented with 1 mM sodium pyruvate and 1x non-essential amino acids (Invitrogen). Caco2 cells were maintained in Eagle's Minimum Essential Medium (EMEM, ATCC) with 20% FBS. Primary human astrocytes and hepatocytes (ScienCell) were cultured in their respective growth media provided by ScienCell. Primary human macrophages were cultured in RPMI-1690 media (Gibco) with 10% FBS.

For immunoblotting, mRNA, ELISA and cell viability analyses, CCF-STTG1 cells were seeded in 12-well (400,000 cells/well) or 24-well (200,000 cells/well) plates in growth media. After 24 h, cells were treated with treatment media (1:1 DMEM:F12 with 1% penicillin/streptomycin and 1% FBS) containing DMSO, positive control GW3965, or test compounds for 48 h. Cell lines were seeded in 12-well plates (MEF: 80,000 cells/well; Caco2: 200,000 cells/well; HepG2: 400,000 cells/well; BV2: 80,000 cells/well). After 24 h, cells were washed with serum-free treatment media (1:1 DMEM:F12 with 1% penicillin/streptomycin) and treated in the same media with controls and test compounds for 24 h (MEF) or 48h (Caco2, HepG2, BV2). Primary human astrocytes were seeded in astrocyte growth media using 6-well plates (750,000 cells/well) for native PAGE analysis and Sudan Black B staining, or 12-well plates (300,000 cells/well) for ELISA, qPCR and SDS-PAGE analyses. After 24 h, human astrocytes were treated with test compounds in their regular growth media for 96 h. Primary human macrophages were seeded at approximately 100,000 cells/well in 24-well plates and treated for 72h in their growth media. Aliquots from the same passage of primary human hepatocytes (5^th^ passage of the original vial purchased) were seeded at 150,000 cells/well in 12-well plates, followed by a 48h treatment with test compounds in their growth media.

For all experiments, the final concentration of the vehicle control DMSO was equalized for all treatment conditions. ApoE in cell supernatants was measured by ELISA and total cholesterol content by Amplex Red following centrifugation of supernatants at 14,000 *g* for 1 min. Cellular apoE mRNA and protein expression levels were determined by quantitative RT-PCR and immunoblotting, respectively.

### Cell Viability Assay

CCF-STTG1 cell viability was evaluated using CellTiter Blue (resazurin assay; Promega) in a 12-well plate format 48 h following treatment as described above. Cells were incubated with CellTitre Blue as per the manufacturer’s protocol for 1 h and fluorescence was recorded (560_Ex_/590_Em_) using an Infinite M1000 PRO microplate reader (TECAN). Percent viability is expressed as test compound relative to DMSO control (which was set to 100%).

### ApoE ELISA

The apoE ELISA used for the high throughput screen has been described previously [61]. Briefly, ELISA plates (384-well, Thermo Scientific) were coated with anti-apoE capture antibody (Abcam, cat # ab7620) at 2.5 μg/mL in PBS at 4°C overnight, washed four times with 100 μL wash buffer (0.05% Tween 20 in PBS) using a plate washer (BioTek Instruments), and blocked for 1 h at room temperature (RT) using 1% BSA in PBS (blocking buffer) dispensed with a Microfill microplate dispenser (BioTek Instruments). Media samples or standards (25 μL) were added using a Biomek FX laboratory automated workstation and incubated for 1.5 h at RT. After washing four times as above, 25 μL of 500 ng/mL anti-apoE detection antibody (Abcam, cat # ab20261) in blocking buffer was added, incubated for 1 h at RT, and washed four times as above. Peroxidase substrate (TMB, Sigma-Aldrich, 25 μL) was then added, incubated for 15 min in the dark, followed by adding 25 μL of stop reagent (TMB substrate) using a Wellmate Dispenser. Plates were immediately read at 450 nm absorbance.

For subsequent validation experiments, a different ELISA assay was used. ELISA plates were coated with anti-human apoE mAB E276 antibody (MabTech, cat # 3712–3) at 1.55 μg/mL in PBS at 4°C overnight, washed two times with 150 μL/well PBST (0.05% Tween 20 in PBS), and blocked for 1 h at RT using 0.1% Blocker A (Meso Scale Discovery) in PBST. After 1 h incubation at RT and two washes with PBST, 100 μL of cell supernatant samples, diluted 1:2 in PBST, or human recombinant ApoE standard (MabTech, cat # 3712–10) were added to each well. The plates were incubated for 1 h at RT and washed twice with PBST. Biotinylated anti-human apoE monoclonal antibody E887 (MabTech, cat # 3712-6-250) was added to each well at a concentration of 0.5 μg/mL in blocking buffer, and the plates were incubated for 1 h at RT. After washing four times with PBST, QuantaBlue Substrate (Pierce, cat # 15162) working solution (9 parts of Substrate Solution to 1 part Stable Peroxide Solution) was added to each well, followed by a 15 min incubation at RT. Fluorescence was read on an EnSpire 2300 Multilabel Plate Reader (325_Ex_/420_Em_).

### Cholesterol assay

Cholesterol content in CCF-STTG1 cell supernatants was measured using the Amplex Red Cholesterol assay kit from Molecular Probes (ThermoFisher) as per the manufacturers instructions. Signal from assay media was subtracted from all sample readings.

### Electrophoresis and Immunoblotting

For native PAGE, media samples were mixed with non-denaturing loading dye to a final concentration of 0.04% bromophenol blue, 4.0% glycerol, and 100 mM Tris (pH 6.8) and resolved on 6% non-denaturing Tris-HCl polyacrylamide gels. Media samples were concentrated 7x for immunoblotting and 15x for Sudan Black B staining using Vivaspin 15R centrifugal concentrators with a molecular weight cutoff of 10,000 Da (Sartorius Stedim Biotech). To visualize apoE, native gels were transferred as described below and probed with 1:2000 anti-apoE antibody (Meridian, cat# K74190G). To visualize lipids, native gels were stained in 0.4% (w/v) Sudan Black B (Sigma) dye solution containing 16% acetone and 12% acetic acid overnight and destained with 100% acetic acid for 20 min prior to transferring to an 0.8% acetic acid and 4% methanol solution for imaging. Gel images were captured using a Bio-Rad ChemiDoc MP Imaging System (Bio-Rad).

To prepare cell lysates, cells were washed with 1x PBS and lysed in radioimmunoprecipitation assay (RIPA) lysis buffer (20 mM Tris, 1% NP40, 5 mM EDTA, 50 mM NaCl, 10 mM Na pyrophosphate, 50 mM NaF, and complete protease inhibitor (Roche), pH 7.4). RIPA cellular lysates were sonicated at 30% output for 10 s. Protein concentration was determined by BCA protein assay (Pierce). Cellular proteins (20–40 μg/well) were mixed with loading dye containing 2% SDS, 1% ß-mercaptoethanol, and 5 U/μL DNaseI (Invitrogen). Samples were incubated for 5 min at 90°C and resolved on 10% Tris-HCl polyacrylamide gels. Proteins were transferred onto polyvinylidene difluoride (PVDF, Millipore) membranes at 24 V overnight at 4°C. After blocking with 5% non-fat milk in PBS for 1 h, membranes were probed overnight at 4°C with 1:2000 goat-anti-human apoE (Chemicon, cat# AB947), 1:1000 goat anti-murine apoE (Santa Cruz, cat# sc-6384), 1:2000 monoclonal anti-ABCA1 (a gift from Dr. Michael Hayden [62]), 1:2000 goat-anti-human LDLR (R&D Systems, cat# AF2148) or 1:5000 anti-β-actin. Membranes were washed with 2x PBST (2x PBS with 0.05% Tween-20) four times for 7 min each and incubated for 1 h with horseradish peroxidase (HRP)-labeled anti-goat (1:1000) or anti-mouse (1:1000 for ABCA1 detection, 1:5000 for actin detection) secondary antibodies (Jackson Immuno-Research). Results were visualized using chemiluminescence (ECL, Amersham). Films were scanned and band density was quantified using ImageJ software (version 1.47q, National Institutes of Health). Levels of ABCA1, apoE and LDLR were normalized to β-actin.

### Capillary Western blotting by Wes Simple Western

ABCA1 levels in primary human astrocytes were assessed using an automated Simple Western system (ProteinSimple) [63, 64]. In brief, proteins are separated by size-based capillary electrophoresis similar to conventional SDS-PAGE. After protein separation, the instrument performs automated immunoblotting by first incubating with primary antibody followed by HRP-conjugated secondary antibody. Detection with luminol/hydrogen peroxide yields a chemiluminescent signal. All procedures were performed with manufacturer’s reagents, except for primary antibodies, according to the user manual. Samples were loaded at a protein concentration of 0.5 μg/μL, incubated with primary antibodies (ABCA1 1:5000; GAPDH 1:10,000) for 60 min and secondary anti-mouse IgG for 30 min. Data were analyzed with Compass software (ProteinSimple). ABCA1 levels were normalized to GAPDH.

### Quantitative RT-PCR

Cells were first lysed in Trizol (Invitrogen). RNA was extracted and treated with DNase I according to the manufacturer’s protocol (Invitrogen). cDNA was generated using oligo-dT primers and Taqman reverse transcription reagents (Applied Biosystems). Quantitative real-time PCR primers (Table 1) were designed using Primer Express (Applied Biosystems) to span human- or murine-specific regions of apoE, ABCA1, LXRα, IDOL and SREBP-1c. Real-time quantitative PCR was done with SYBR Green reagents (Applied Biosystems) on a StepOne Plus (Applied Biosystems). Each sample was assayed at least in duplicate, normalized to GAPDH for human cells or β-actin for murine cells, and relative gene expression analyzed (using DMSO as the study calibrator) using a fold-change threshold of > 1.5.

**Table 1:** Quantitative RT-PCR primer sequences.

Gene	Forward primer (5’ to 3’)	Reverse primer (5’ to 3’)
**Human *APOE***	CTGCTCAGCTCCCAGGTC	TTGTTCCTCCAGTTCCGATT
**Human *ABCA1***	AAGCACTTCCTCCGAGTCAA	CTGTCCTTGGCCAGCTTTAG
**Human *NR1H3* (LXRα)**	GGTACAACCCTGGGAGTGAG	TGGGGTTGATGAATTCCACT
**Human *MYLIP* (IDOL)**	AAGTTCTTCGTGGAGCCTCA	CTCTGGGGAACACAAGAGG
**Human *SREBFI* (SREBP-1c)**	GGAGCCATGGATTGCACATT	GGCCCGGGAAGTCACTGT
**Human *GAPDH***	CCTGCACCACCAACTGCTTA	CATGAGTCCTTCCACGATACCA
**Murine *Actinb***	ACGGCCAGGTCATCACTATTG	CAAGAAGGAAGGCTGGAAAAGA
**Murine *apoE***	AACCGCTTCTGGGATTACCT	TGTGTGACTTGGGAGCTCTG
**Murine *Abca1***	CTGACCTATGTGCTGCCGTA	GAGCCGGTCATCAATCTCAT
**Murine *Srebpf1* (SREBP-1c)**	ATCGGCGCGGAAGCTGTCGGGGTAGCGTC	ACTGTCTTGGTTGTTGATGAGCTGGAGCAT

### Insecticide assay

Cabbage looper (*Trichoplusia ni*) eggs of a pesticide-susceptible strain were obtained from the Great Lakes Forestry Centre, Natural Resources Canada (Sault Ste. Marie, ON, Canada). The colony was maintained on a pinto bean-based artificial diet (Bio-Serv Inc., Frenchtown, NJ, USA) at 23–26°C and a 16:8 h LD photoperiod. Insecticidal activities of 82879 and the positive control permethrin were evaluated via topical application [65]. In brief, each of ten third instar larvae received 1 μL of DMSO solution of the test compounds or DMSO alone as the negative control using a Hamilton Microliter syringe attached to a repeating dispenser. Two concentrations of 82879 (0.5 mM, 1.25 mM) and one of permethrin (0.5 mM) were tested. Treated larvae were transferred into a 7-cm-diameter glass Petri dish with a piece of artificial diet (0.5 g) and kept under the same conditions as above. Mortality was recorded after 24 h. If no movement was observed when the insect was probed with a fine brush, then a larva was considered dead.

### Nuclear Receptor Transactivation Assays

To evaluate direct agonist activities against human LXRα, LXRβ, RXRα, RXRβ, RXRγ, PPARγ and FXR, Chinese hamster ovary cell-based nuclear receptor reporter assays were conducted by Indigo Biosciences (State College, PA). Briefly, LXRα, LXRβ, RXRα, RXRβ, RXRγ, PPARγ or FXR luciferase reporter cells were plated into 96-well assay plates and treated in triplicate with test compounds for 24 h prior to quantifying nuclear receptor agonist activity. The final DMSO concentration was 0.1%. Cells were also tested with their respective positive control agonists (LXRα and LXRβ: T0901317; RXRα, RXRβ, and RXRγ: 9-*cis*- Retinoic Acid; PPARγ: Rosiglitazone; FXR: GW4064).

### Statistics

Data are shown as mean ± SD (for measured concentrations) or mean ± 95% confidence interval (CI, for fold-change analyses) of the indicated number of independent experiments. Except for mRNA analysis where ΔC_T_ values were used, linear raw data were first log transformed and then analyzed by ANOVA with a blocking factor (Experiment) and with a Dunnett’s multiple comparison post-test. For the LXR-dependency study, the Linear Mixed Model with a Sidak's multiple-comparison test on ΔCT values were used to compare between DMSO and test compound treatment within each genotype, whereas ANOVA with a blocking factor (Experiment) were performed on ΔΔC_T_ values (ΔC_T_ of test compound - ΔC_T_ of DMSO control) to determine the difference of the magnitude of drug effect between two genotypes. All statistical analyses were performed using SPSS (version 19 and 21) and P-values <0.05 were considered significant. Prism 5 (GraphPad Software) was used to plot all data.

## Results

### High throughput screen identifies compound 82879 as a novel apoE modulator in CCF-STTG1 astrocytoma cells

**Fig 1:**
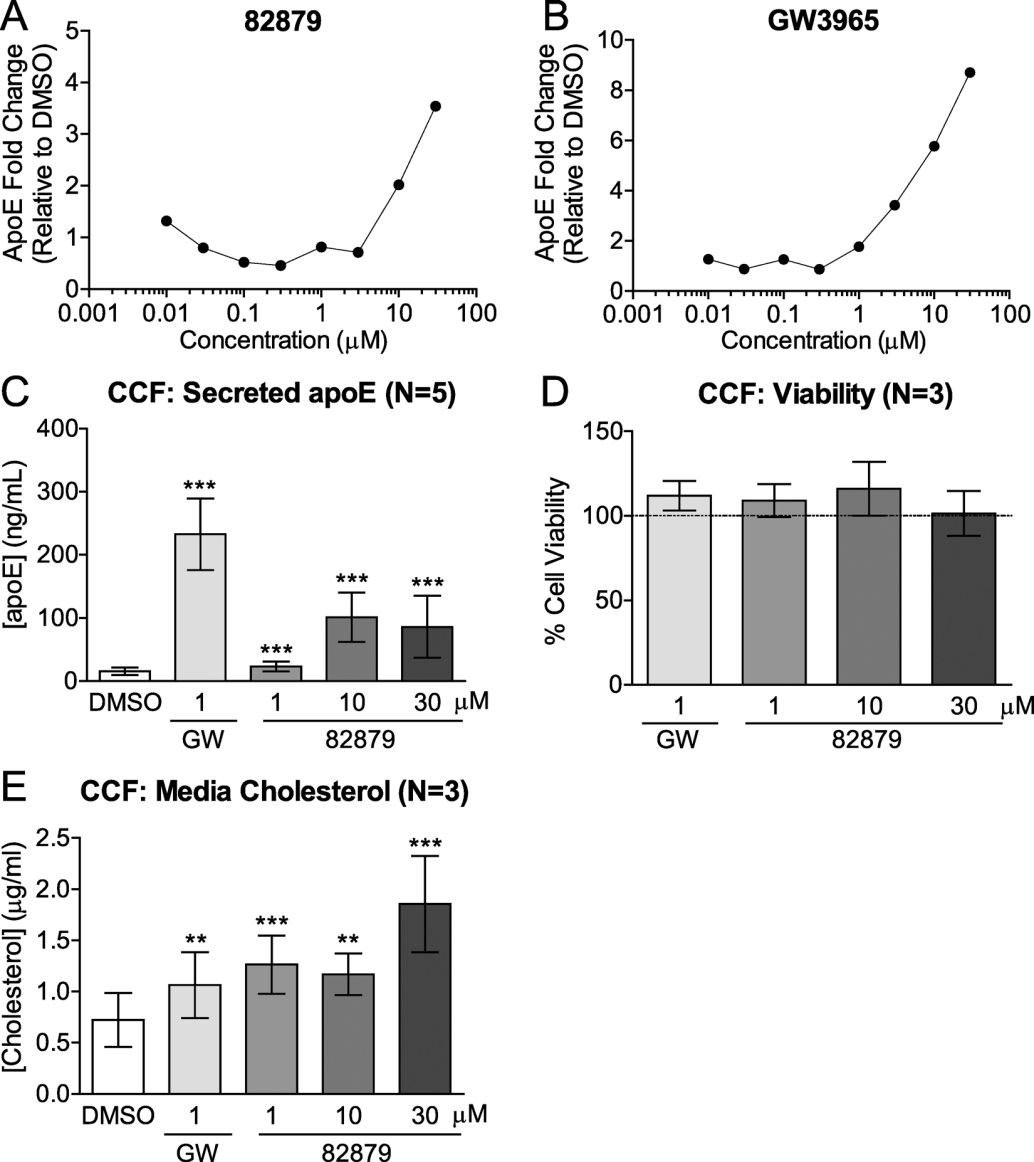
Compound 82879 increases secreted apoE without affecting cell viability in CCF-STTG1 cells. CCF-STTG1 (CCF) astrocytoma cells were treated with (*A*) compound 82879 or (*B*) positive control GW3965 and media apoE was measured following 96 h of treatment (0.1 to 30 μM) using the initial screen assay conditions. Data are expressed as fold-change relative to DMSO treatment (N = 1). CCF cells were treated with DMSO alone, GW3965 (GW), or compound 82879 for 48 h prior to measuring (*C*) media apoE (*D*) cell viability (expressed as fold-change relative to DMSO treatment, which corresponds to 100% on the Y-axis, dashed line) and (*E*) total media cholesterol content. Graphs represent mean and SD from N independent experiments indicated in brackets. Statistics were determined by a one-way ANOVA with a blocking factor (Experimental) and a Dunnett’s post-test (*** p< 0.001).

We performed a high-throughput screen to identify compounds from a library of 104,000 small molecules that enhance apoE secretion from CCF-STTG1 cells following 96 h of treatment. 318 compounds passed a fold-change threshold of 2 for apoE induction in the orthogonal screening approach and a single positive hit, compound 82879, was subsequently confirmed to enhance apoE secretion in a concentration-responsive fashion as a single compound under the same assay conditions (Fig 1A and 1B). We then validated the results from the initial screen in independent assays using a different assay format and an abbreviated 48 h time point. Following exposure to either compound 82879 or the LXR agonist GW3965 as a positive control, we observed significantly increased secreted apoE levels at 10 μM and 30 μM of 82879, up to 6-fold greater than vehicle control, confirming the initial screen result where these concentrations yielded at least a 2-fold induction over baseline (Fig 1C). This increase in media apoE was similar, albeit at a lower magnitude of response, to GW3965. Importantly, we observed no change in cell viability at any of the concentrations tested (Fig 1D). To determine if increased apoE secretion was associated with increased media lipid content, total cholesterol was measured in CCF-STTG1 cell supernatants and was significantly increased in both GW3965 and 82879 treated cells (Fig 1E).

### The activity of 82879 is distinct from structurally related molecules including pyrethroid ester insecticides

**Fig 2:**
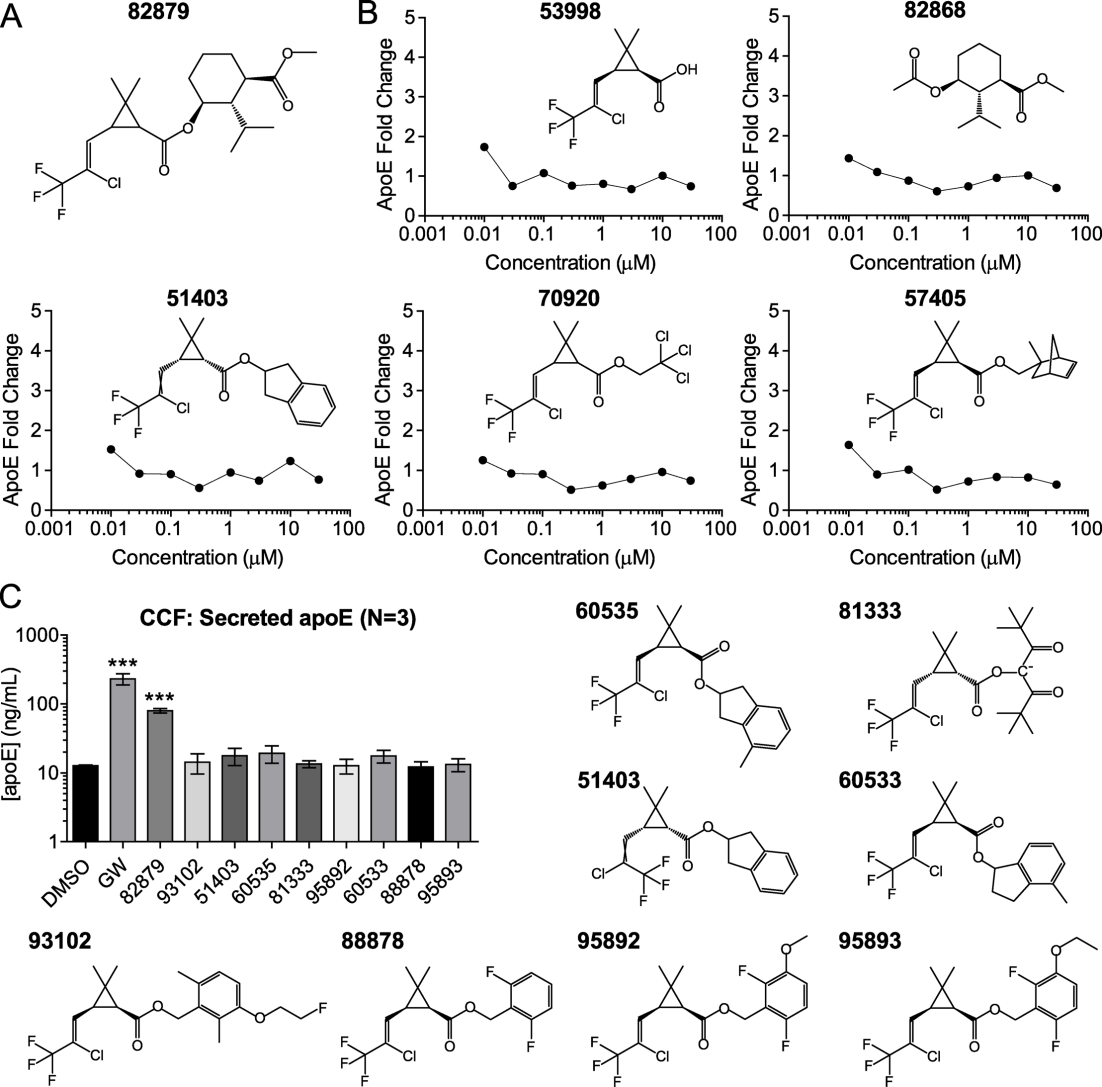
Structural analogues of 82879 do not increase secreted apoE in CCF-STTG1 cells. (*A*) Planar structure of compound 82879. (*B*) Structural analogues from the small molecule library used for the high throughput screen were tested for their effects on apoE secretion. Media apoE was measured following 96 h of treatment (0.1 to 30 μM) using the initial screen assay conditions. Data are expressed as fold-change relative to DMSO treatment (N = 1). (*C*) Additional structural analogues were tested at a concentration of 10 μM and apoE secretion was measured following 48 h of CCF-STTG1 (CCF) cell treatment using the validation assay conditions (N = 3). Graphs represent mean and SD of apoE concentration. Statistics were determined by one-way ANOVA with blocking factor (Experiment) and a Dunnett’s post-test (*** p<0.001).

**Fig 3:**
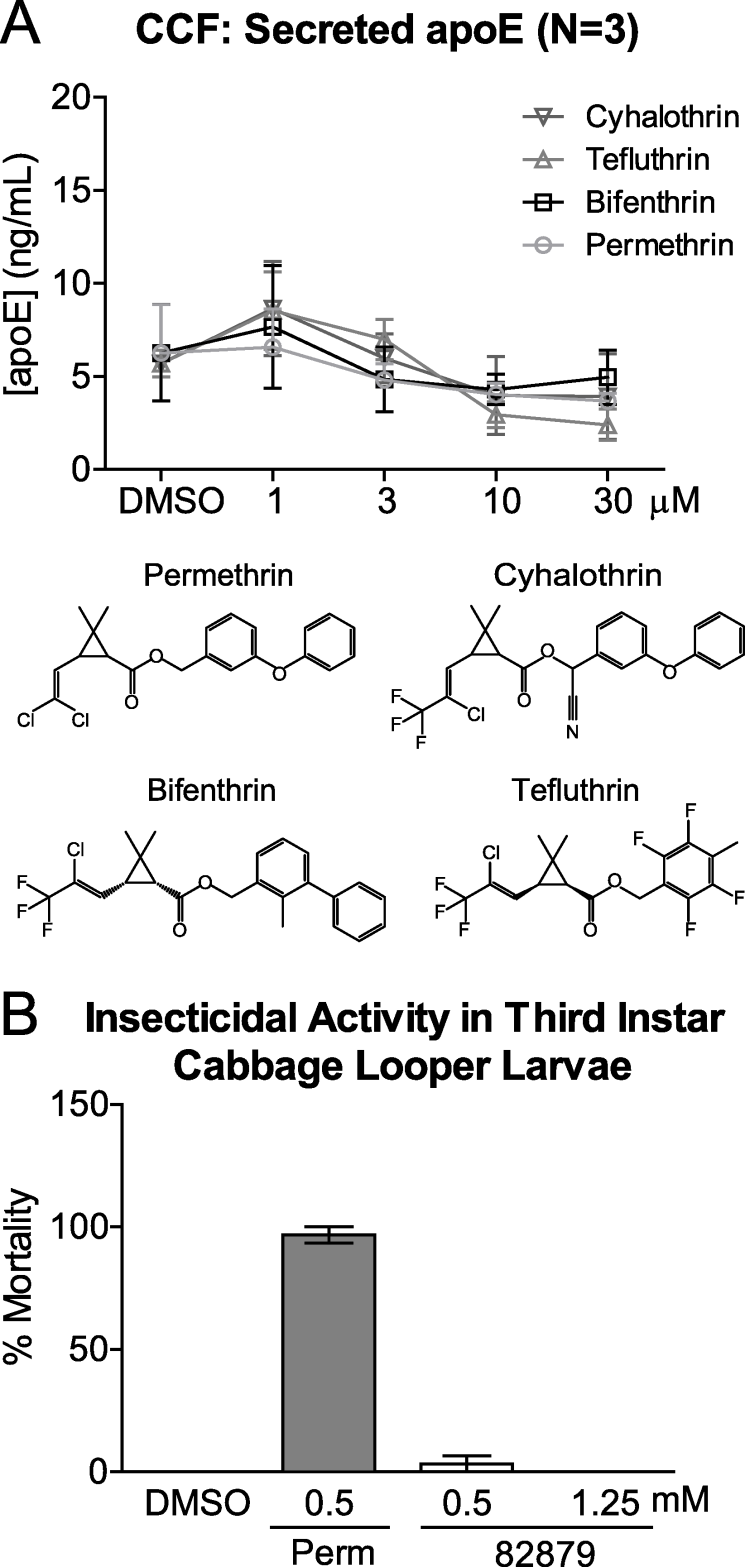
Distinct activity of 82879 compared to pyrethroid ester insecticides. (*A*) CCF-STTG1 (CCF) astrocytoma cells were treated with DMSO, 1 μM of GW3965, or 1–30 μM each of permethrin, bifenthrin, tefluthrin, cyhalothrin for 48 h prior to measurement of media apoE. Graphs represent mean and SD of apoE concentration from three independent experiments. Statistics were determined by a one-way ANOVA with a blocking factor (Experiment) and Dunnett’s post-test. (*B*) Cabbage looper (*Trichoplusia ni*) larvae were topically treated with 1 μL of 0.5 mM (n = 30) or 1.25 mM (n = 15) 82879, or 0.5 mM permethrin (Perm, n = 30) as a positive control or DMSO (n = 30) as a negative control. Mortality was recorded after 24 h. If no movement was observed when the insect was probed with a fine brush, then a larva was considered dead. No mortality was observed in the DMSO control.

Compound 82879 is a chrysanthemic ester (Fig 2A, racemic mixtures of unknown proportions) similar to pyrethroid esters that have been developed for commercial use as insecticides. A key difference is that compound 82879 contains a saturated cyclohexyl ring rather than an unsaturated moiety found in pyrethroid insecticides. To test whether apoE upregulation is a feature common to this class, we explored the structure-activity relationship (SAR) around 82879 by measuring apoE activity derived from only the acid (compound 53998) or alcohol (compound 82868) moieties of 82879 or by evaluating pyrethroid esters where the structure of the acid moiety was fixed to match that of 82879 albeit with a certain degree of stereoisomerism (Fig 2). Using the initial screen assay format and 5 analogues (acid moiety, alcohol moiety and 3 pyrethroids) from the original screening library, we confirmed that these structurally similar compounds showed no activity in the apoE secretion assay over a 0.1 to 30 μM 8-point dose response curve (Fig 2B). Subsequently, we tested an additional 8 pyrethroids from the original screening library with shared structural features at a single 10 μM concentration using the validation assay format. These compounds were also negative for apoE secretion (Fig 2C). We then expanded our SAR panel to marketed pyrethroids, including permethrin, the first synthetic pyrethroid insecticide with sufficient photostability for agricultural use [66]. Permethrin, bifenthrin, tefluthrin, and cyhalothrin, which share the halogenated chrysanthemic acid moiety with compound 82879, had no effect on secreted apoE levels when tested in a 4-point dose response assay at concentrations from 1 to 30 μM (Fig 3A), doses that did not affect cell viability (supplementary data in S1 Dataset).

We next tested 82879 in an insecticide assay using third instar larvae of the cabbage looper caterpillar, *Trichoplusia ni*, a model species for testing botanical insecticides. Larvae were topically treated with 1 μL of 0.5 mM or 1.25 mM 82879 or 0.5 mM permethrin as a positive control. Compound 82879 resulted in a single dead larvae at 0.5 mM, and no mortality at 12.5 mM which is within the range of mortality for the controls, compared to >95% mortality with permethrin at 0.5 mM (Fig 3B). Together, these results suggest that the mechanism of action of the active moiety(ies) in 82879 with respect to both promoting apoE modulation and lack of insecticidal activity is distinct from the known mechanism of action of pyrethroid insecticides.

### 82879 upregulates apoE and other LXR target genes in CCF-STTG1 cells

**Fig 4:**
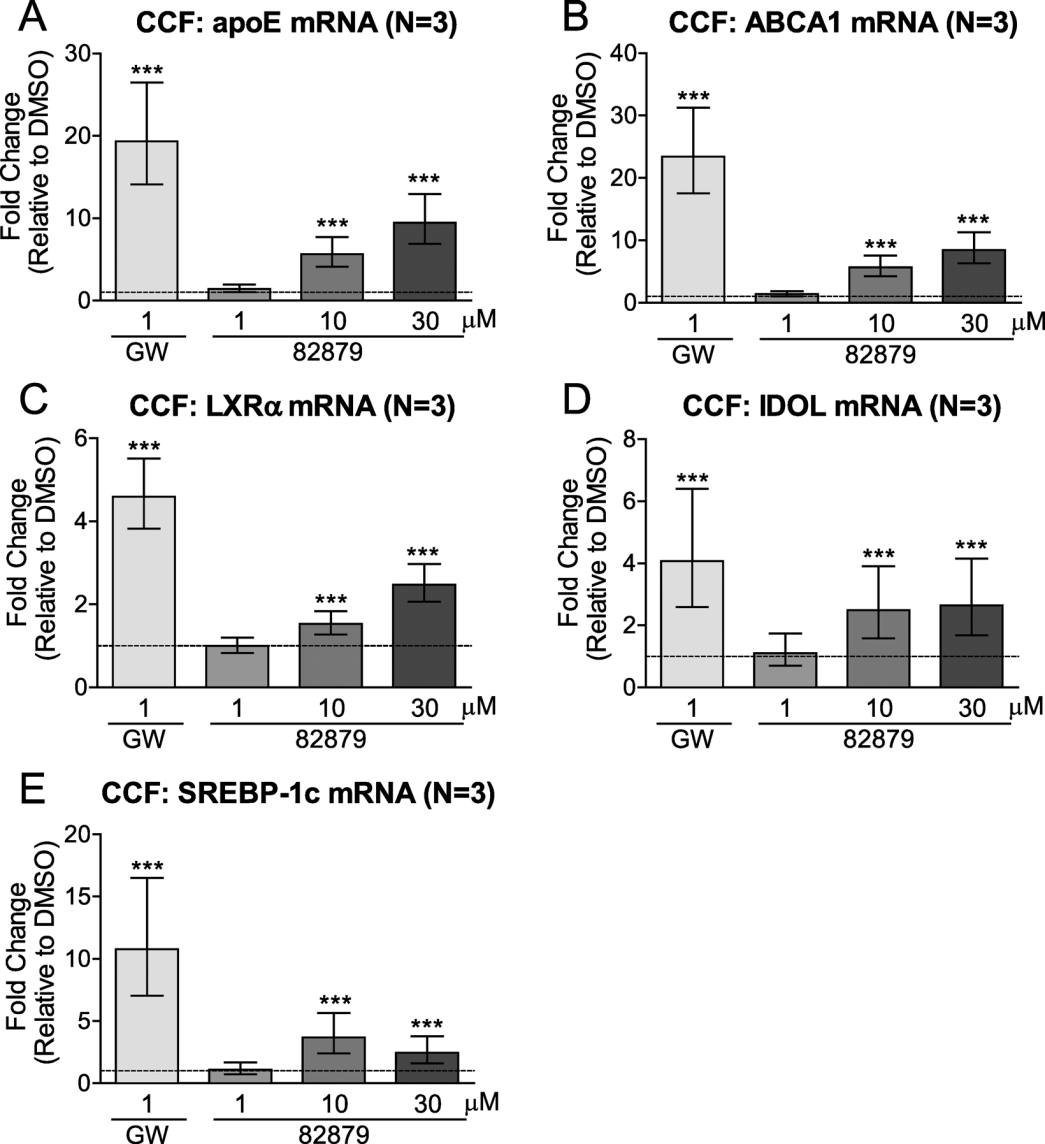
Compound 82879 upregulates LXR target gene expression in CCF-STTG1 cells. CCF-STTG1 (CCF) astrocytoma cells were treated for 48 h with DMSO alone, GW3965, or compound 82879. Following treatment, the mRNA levels for (*A*) apoE, (*B*) ABCA1, (*C*) LXRα, (*D*) IDOL and (E) SREBP-1c were determined in whole cell lysates. Data are expressed as fold-change relative to DMSO treatment (which corresponds to 1 on the Y-axis, dashed line). Graphs represent mean and 95% CI (N = 3). Statistics were determined by a one-way ANOVA with a blocking factor (Experiment) and a Dunnett’s post-test (*** p< 0.001).

As apoE is transcriptionally regulated through the LXR pathway, we tested the ability of 82879 to induce expression of apoE and other LXR-regulated genes (ABCA1, LXRα and IDOL, inducible degrader of LDLR, and SREBP-1c) in CCF-STTG1 cells. Following 48 h of treatment, the positive control LXR agonist GW3965 significantly upregulated apoE, ABCA1, LXRα, IDOL and SREBP-1c as expected (Fig 4). At concentrations of 10 and 30 μM, 82879 induced apoE mRNA up to 9.4-fold, indicating that increased media levels of apoE are in part due to increased apoE transcription (Fig 4A). At these concentrations, we also observed up to 8.4-fold induction of ABCA1 mRNA (Fig 4B). Compound 82879 was able to upregulate LXRα mRNA itself, presumably by leading to positive feedback in LXR signaling activity (Fig 4C). The LXR signaling pathway inhibits LDLR, which mediates apoE uptake and recycling, through the transcriptional upregulation of the E3 ubiquitin ligase IDOL. Compound 82879 also induced IDOL mRNA expression (Fig 4D). Hence apoE retention in media, through downregulated LDLR, may also contribute to the increased total secreted apoE levels. Finally, 82879 also induced SREPB-1c, an LXR target gene involved in fatty acid synthesis, in CCF-STTG1 cells (Fig 4E).

**Fig 5:**
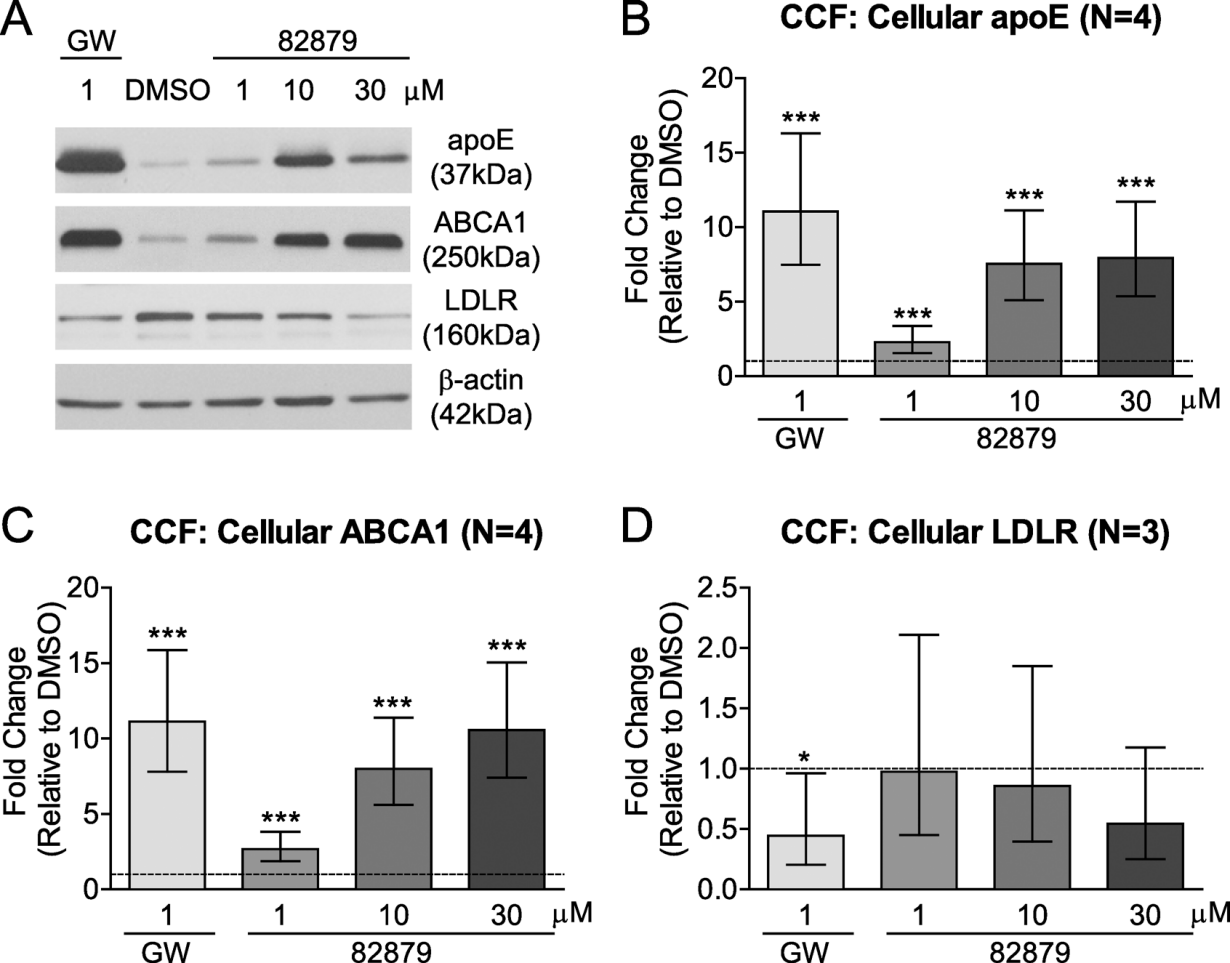
Compound 82879 upregulates apoE and ABCA1 and shows a trend toward downregulated LDLR protein levels in CCF-STTG1 cells. CCF-STTG1 (CCF) astrocytoma cells were treated with DMSO alone, GW3965, or compound 82879 for 48 h. (*A*) A representative Western Blot. Protein levels in whole cell lysates were determined by densitometry for (*B*) apoE (*C*), ABCA1 (*D*), LDLR. Data were normalized over β-actin levels (*A*) and expressed as fold-change relative to DMSO treatment (which corresponds to 1 on the Y-axis, dashed line). Graphs represent mean and 95% CI from N independent experiments indicated in brackets. Statistics were determined by a one-way ANOVA with a blocking factor (Experiment) and a Dunnett’s post-test (* p<0.05, *** p< 0.001).

Using CCF-STTG1 cells under the same assay conditions, we evaluated cellular protein levels and found them to be consistent with the mRNA changes. As expected, GW3965 significantly upregulated cellular apoE and ABCA1 protein. Showing a comparable magnitude of response to GW3965, compound 82879 significantly increased cellular apoE and ABCA1 protein starting at 1 μM, reaching a 7.9-fold increase in apoE and a 10.6-fold increase in ABCA1 protein levels at 30 μM (Fig 5A–5C). We also evaluated LDLR and observed a trend towards decreased LDLR protein levels in cells treated with the highest dose of 82879, similar to the reduction seen in GW3965-treated cells (Fig 5A and 5D) and consistent with significantly increased IDOL expression. Taken together, our results show that comparable to GW3965, 82879 increases both apoE expression and secretion and induces the LXR target genes ABCA1, LXRα, as well as IDOL, which may contribute to the elevated apoE levels in media by inhibiting LDLR-mediated apoE uptake and recycling.

### ApoE induction by 82879 includes both LXRα-dependent and LXRα-independent components

We then evaluated the direct LXR agonism of 82879 in a cell based nuclear receptor agonist assay through a study performed at Indigo Bioscience. We measured potential agonist activities directed against several human nuclear receptors including LXRα, LXRβ, RXRα, RXRβ, RXRγ, PPARγ, and FXR. Compound 82879, which exists as a racemic mixture, was revealed to have modest LXR transactivation, exhibiting agonist activity against LXRα (2.1-fold) and LXRβ (4-fold) at 10 μM (Table 2).

**Table 2:** Nuclear receptor agonist activities of compound 82879.

Compound	μM	Fold-Activation						
		FXR	LXRα	LXRβ	PPARγ	RXRα	RXRβ	RXRγ
**DMSO**	0.10%	1.0	1.0	1.0	1.0	1.0	1.0	1.0
**82879**	1	1.4	1.1	1.0	1.1	1.0	1.3	0.8
	10	1.7	**2.1**	**4.0**	1.2	1.2	1.5	0.8

Agonist data values ≥ 2-fold activation are bolded.

**Fig 6:**
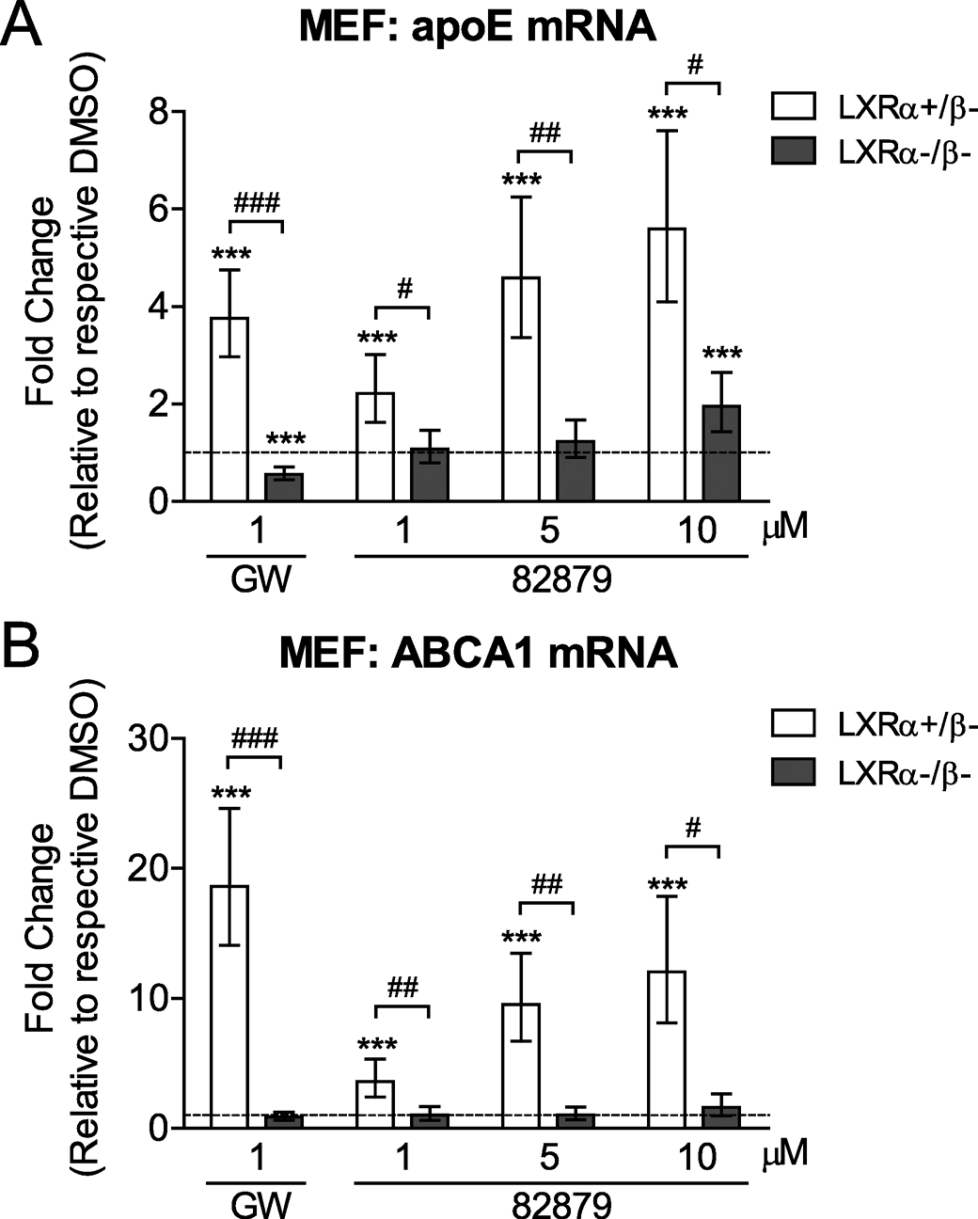
82879 induces LXRα-dependent upregulation of ABCA1 mRNA in MEFs whereas apoE expression is stimulated through LXR-dependent and LXR-independent activities. LXRα-/β-, and LXRα expressing (LXRα+/β-) MEF cells were treated for 24 h withDMSO alone, GW3965, or compound 82879 at the indicated concentrations. Differential mRNA expression levels of (*A*) apoE and (*B*) ABCA1 were measured. Data are expressed as fold-change relative to the respective control group (dashed line) within each genotype. The responses to drug treatment compared to the DMSO control within each genotype (***p <0.001) were compared using a Linear Mixed Model with a Sidak's multiple-comparison test on ΔCT values obtained by qRT-PCR. The magnitudes of drug effect between two genotypes were compared using one-way ANOVA with a blocking factor (Experiment) on ΔΔCT values (# p<0.05, ## p<0.01, ### p<0.001). Data are expressed as mean and 95% CI from at least 6 independent experiments for GW3965 treatment and 3 experiments for 82879 treatments.

To determine whether 82879 requires LXR to induce apoE and ABCA1 mRNA, we used immortalized mouse embryonic fibroblasts (MEFs) deficient in both LXRα and LXRβ and isogenic MEFs that were reconstituted with LXRα [59]. MEFs were treated for 24 h with 1, 5, 10 μM 82879 or 1 μM GW3965 (Fig 6). While induction of apoE mRNA by 1 μM GW3965 and 82879 at 1 and 5 μM was completely abolished in LXRα-/β- MEFs, 10 μM 82879 could still significantly elicit apoE upregulation albeit at a reduced level in the double knockout cells, suggesting the involvement of an LXR-independent pathway (Fig 6A). Conversely, 82879-mediated ABCA1 mRNA induction was entirely LXR-dependent, as LXRα-/β- MEFs were non-responsive to 82879 even at 10 μM (Fig 6B). Together, our data suggest that although 82879 displays modest direct LXR agonism and that 82879-mediated upregulation of ABCA1 mRNA is entirely LXR-dependent, 82879 can also stimulate apoE expression using LXR-independent mechanisms.

### 82879 regulates LXR target genes in a cell-type-specific manner

**Fig 7:**
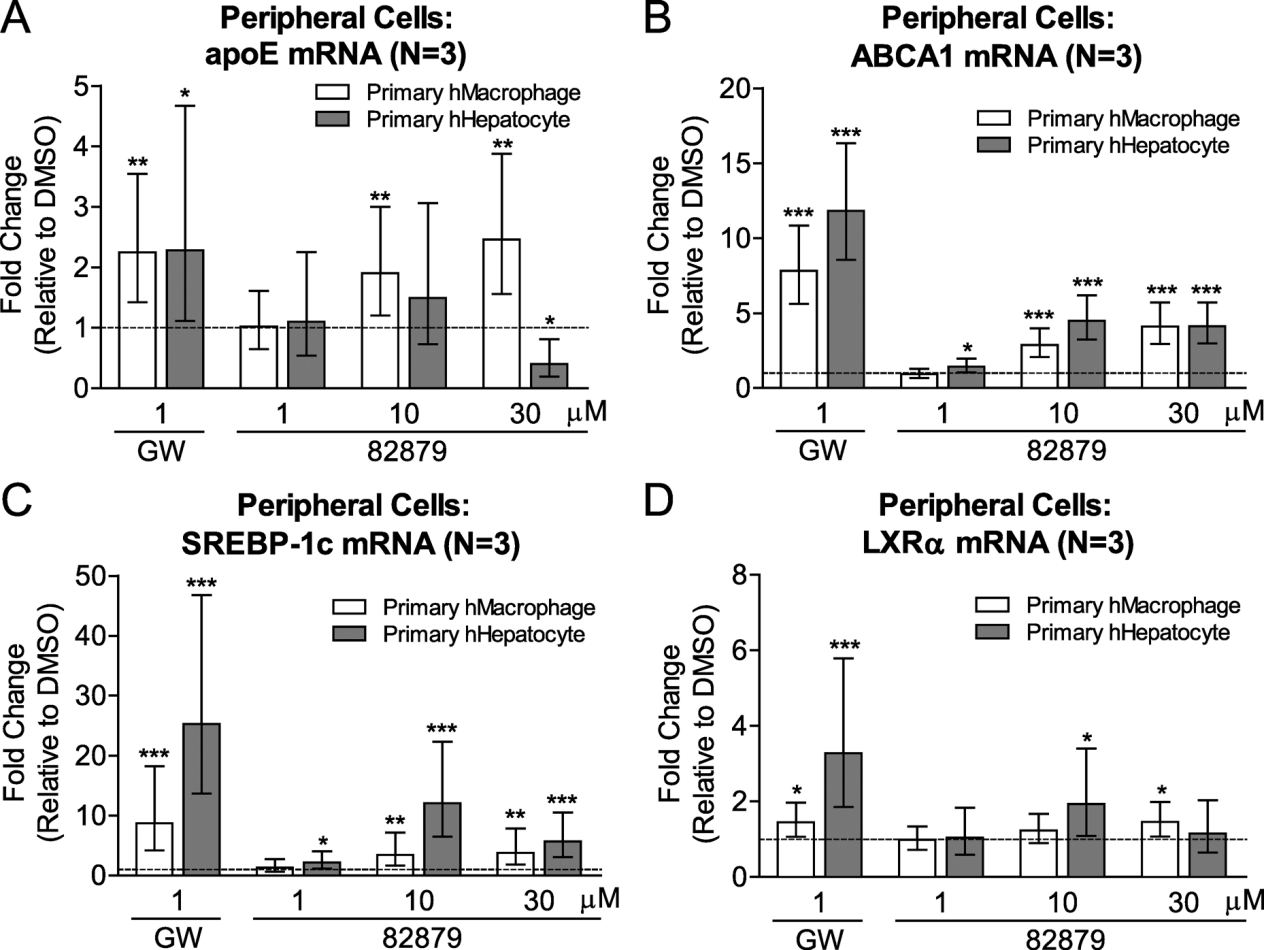
82879 regulates LXR target genes differentially in primary human macrophages and hepatocytes. Primary human macrophages (hMacrophage) and hepatocytes (hHepatocytes) were treated for 48h with DMSO alone, GW3965, or compound 82879. Following treatment, the mRNA levels for (*A*) apoE, (*B*) ABCA1, (*C*) SREBP-1c, and (*D*) LXRα were determined in whole cell lysates. Data are expressed as fold-change relative to DMSO treatment (which corresponds to 1 on the Y-axis, dashed line). Graphs represent mean and 95% CI of 3 independent experiments. Statistics were determined by a one-way ANOVA with a blocking factor (Experiment) and a Dunnett’s post-test (* p<0.05, ** p<0.01, *** p< 0.001).

We next explored the responses to 82879 in peripheral cells including primary human macrophages and hepatocytes. Intriguingly, while 82879 upregulated apoE mRNA in a dose dependent manner in macrophages, 82879 not only failed to induce apoE expression in human hepatocytes but rather downregulated apoE mRNA at 30 μM (Fig 7A). ABCA1 mRNA induction also followed a similar dose dependent manner in macrophages, whereas ABCA1 upregulation in hepatocytes plateaued at 10 μM (Fig 7B). Compound 82879 also elevated other LXR target genes, SREBP-1c and LXRα, in both macrophages and hepatocytes to various degrees at different doses (Fig 7C and 7D).

In addition to primary human macrophages and hepatocytes, we also tested 82879 in additional human and murine cell lines including HepG2, Caco2 and BV2. As summarized in Table 3, compound 82879 failed to induce apoE mRNA in all the other cell lines tested. BV2 cells responded with a statistically significant 1.8-fold increase of ABCA1 mRNA to 10μM 82879, whereas the same dose resulted a 2.9-fold increase in SREBP-1c mRNA in Caco2 cells. Overall, these cell lines were not responsive to compound 82879 with respect to LXR target gene activities.

**Table 3:** Response of LXR target genes to compound 82879 in various cell lines.

Cell Line	**ApoE mRNA Fold Change over DMSO**					
	1μM GW3965			10μM 82879		
	Mean FC	95% CI [UB, LB]	P value	Mean FC	95% CI [UB, LB]	P value
HepG2	**1.5**	[2.1, 1.1]	**0.010**	1.0	[1.4, 0.8]	0.997
Caco2	0.8	[1.1, 0.6]	0.164	0.9	[1.2, 0.6]	0.426
BV2	**2.2**	[3.8, 1.2]	**0.012**	0.9	[1.7, 0.5]	0.988
Cell Line	**ABCA1 mRNA Fold Change over DMSO**					
	1μM GW3965			10μM 82879		
	Mean FC	95% CI [UB, LB]	P value	Mean FC	95% CI [UB, LB]	P value
HepG2	1.7	[3.4, 0.9]	0.110	0.8	[1.6, 0.4]	0.847
Caco2	**3.1**	[5.9, 1.6]	**0.002**	1.3	[2.5, 0.7]	0.581
BV2	**3.6**	[6.0, 2.1]	**<0.001**	**1.8**	[3.1, 1.1]	**0.026**
Cell Line	**SREBP-1c mRNA Fold Change over DMSO**					
	1μM GW3965			10μM 82879		
	Mean FC	95% CI [UB, LB]	P value	Mean FC	95% CI [UB, LB]	P value
HepG2	**12.7**	[22.9, 7.0]	**<0.001**	1.5	[2.8, 0.9]	0.162
Caco2	**9.2**	[25.9, 3.3]	**0.001**	**2.9**	[8.2, 1.0]	**0.043**
BV2	**6.3**	[26.7, 1.5]	**0.022**	1.6	[6.7, 0.4]	0.609

FC, fold change; CI, confidence interval; UB, upper bound; LB, lower bound. P-values are calculated by ANOVA with a Dunnett’s post-test from 3 independent experiments. Data values with a p<0.05 are bolded.

### Compound 82879 induces apoE and ABCA1 in primary human astrocytes

**Fig 8:**
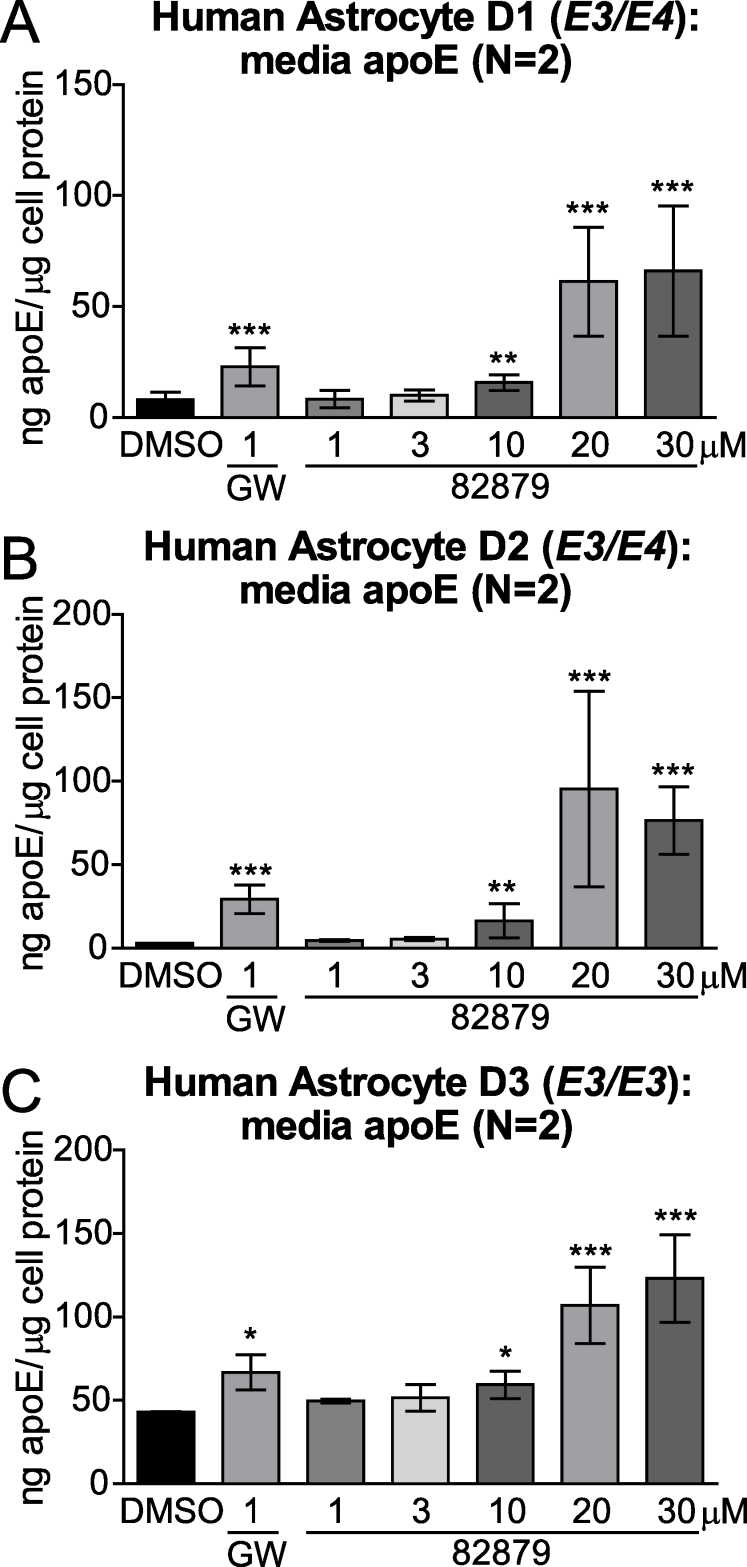
Compound 82879 upregulates apoE secretion in primary human astrocytes. Primary human astrocytes from three donors genotyped for *APOE* (*A*) D1: *E3/E4*, (*B*) D2: *E3/E4*, (*C*) D3: *E3/E3*) were treated with DMSO alone, GW3965, or compound 82879 for 96 h in two independent experiments and apoE secretion levels were measured by ELISA. Graphs represent mean and SD of media apoE concentration normalized against total cellular protein content. Statistics were determined by a one-way ANOVA with a blocking factor (Experiment) and a Dunnett’s post-test (* p<0.05, *** p< 0.001).

**Fig 9:**
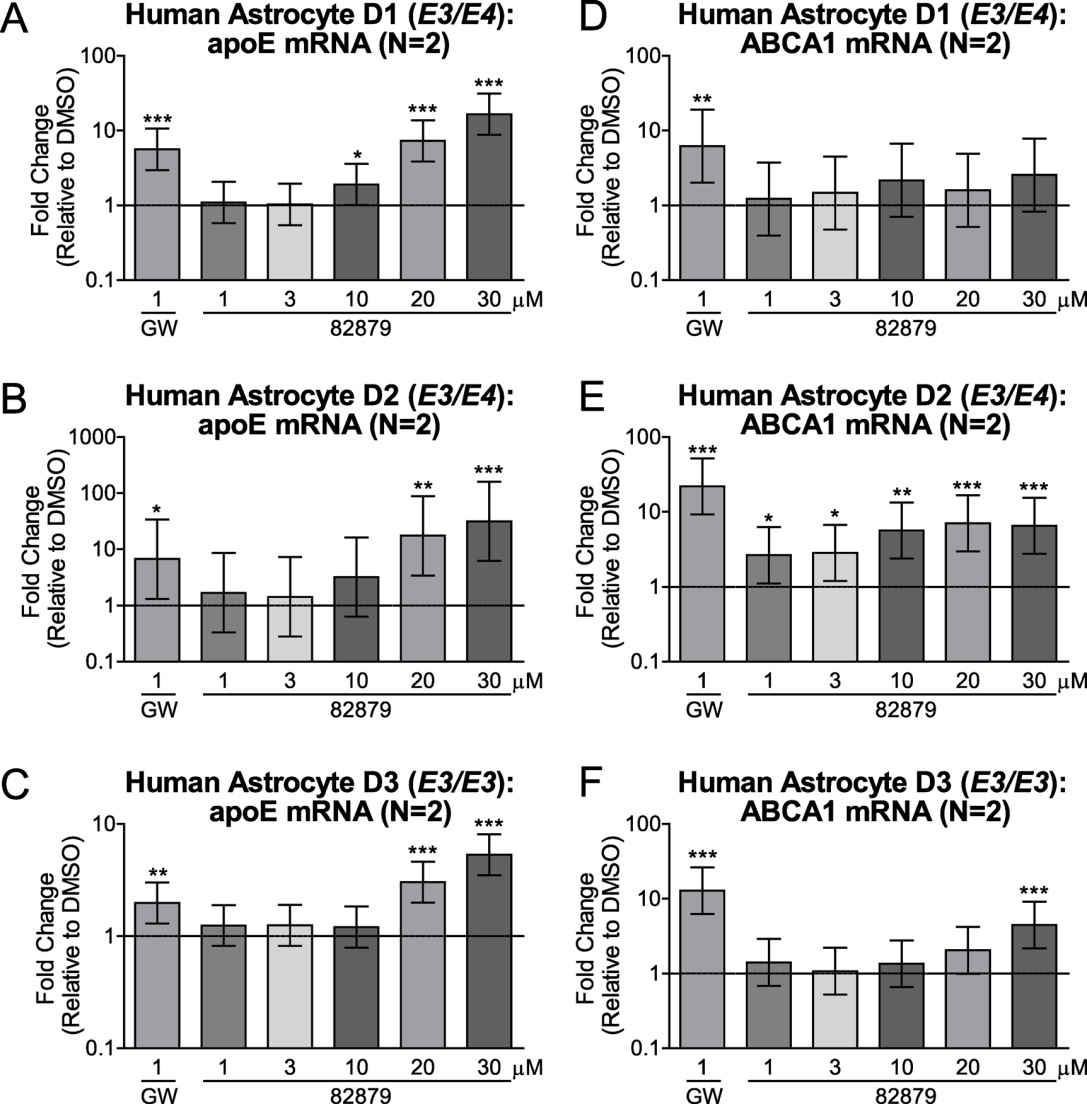
Compound 82879 upregulates apoE and ABCA1 mRNA in primary human astrocytes. Primary human astrocytes from three donors genotyped for *APOE* (D1: *E3/E4*, D2: *E3/E4*, D3: *E3/E3*) were treated with DMSO alone, GW3965, or compound 82879 for 96 h in two independent experiments. (*A-C*) ApoE mRNA and (*D-F*) ABCA1 mRNA levels were measured. Graphs represent mean and 95% CI of fold change relative to DMSO treatment (which corresponds to 1 on the Y-axis, dashed line). Statistics were determined by a one-way ANOVA with a blocking factor (Experiment) and a Dunnett’s post-test (* p<0.05, ** p<0.01, *** p< 0.001).

**Fig 10:**
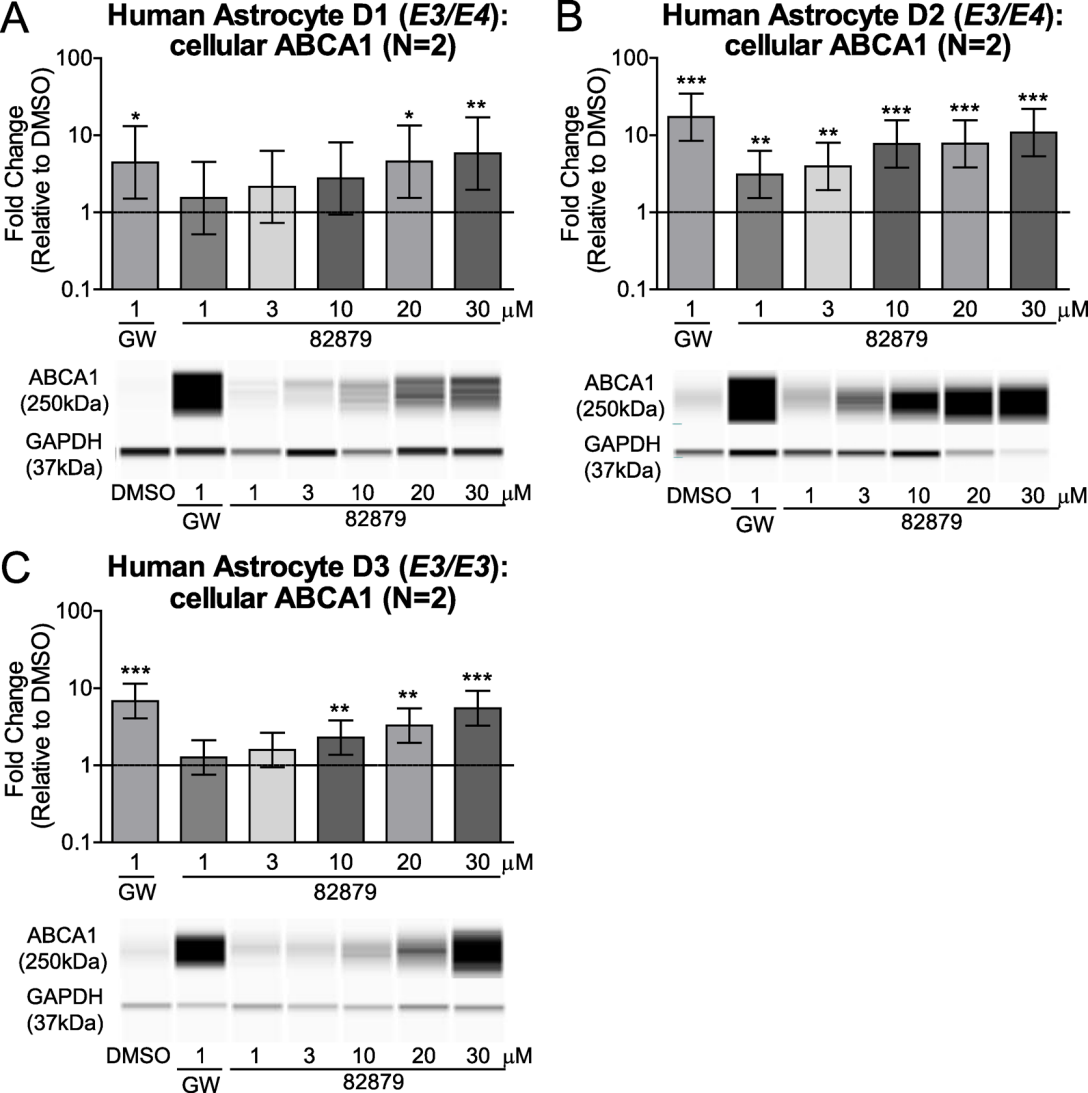
Compound 82879 upregulates cellular ABCA1 protein levels in primary human astrocytes. Primary human astrocytes from three donors genotyped for *APOE* (*A*) D1: *E3/E4*, (*B*) D2: *E3/E4*, (*C*) D3: *E3/E3*) were treated with DMSO alone, GW3965, or compound 82879 for 96 h in two independent experiments. Cellular ABCA1 protein levels were determined by a capillary western system (representative pictures shown). Graphs represent mean and 95% CI of fold change relative to DMSO treatment (which corresponds to 1 on the Y-axis, dashed line). Statistics were determined by a one-way ANOVA with a blocking factor (Experiment) and a Dunnett’s post-test (* p<0.05, ** p<0.01, *** p< 0.001).

To extend our studies beyond CCF-STTG1 astrocytoma cells (*APOE3/APOE4*) [22], we next verified the apoE and ABCA1 activities of compound 82879 in non-transformed primary human astrocytes from three donors genotyped as *APOE3/E3* or *APOE3/E4*. We observed a clear upregulation of apoE secretion in all three donors that plateaued at 20 μM, despite wide variations in both basal apoE levels and the magnitude of apoE induction among different donors, ranging from 2.8-fold up to 30.0-fold (Fig 8A–8C). Compound 82879 also significantly induced apoE mRNA in each respective donor from 5.3-fold up to 31.3-fold (Fig 9A–9C). While ABCA1 transcript expression was significantly elevated following 82879 treatment in two of three donors (Fig 9D–9F), ABCA1 protein levels were significantly increased across all three primary human astrocyte donors (Fig 10). Our data support compound 82879 as a robust inducer of apoE and ABCA1 that validates across two human astrocytic cell types, including CCF-STTG1 astrocytoma cells and more physiologically relevant primary astrocytes from three distinct donors.

**Fig 11:**
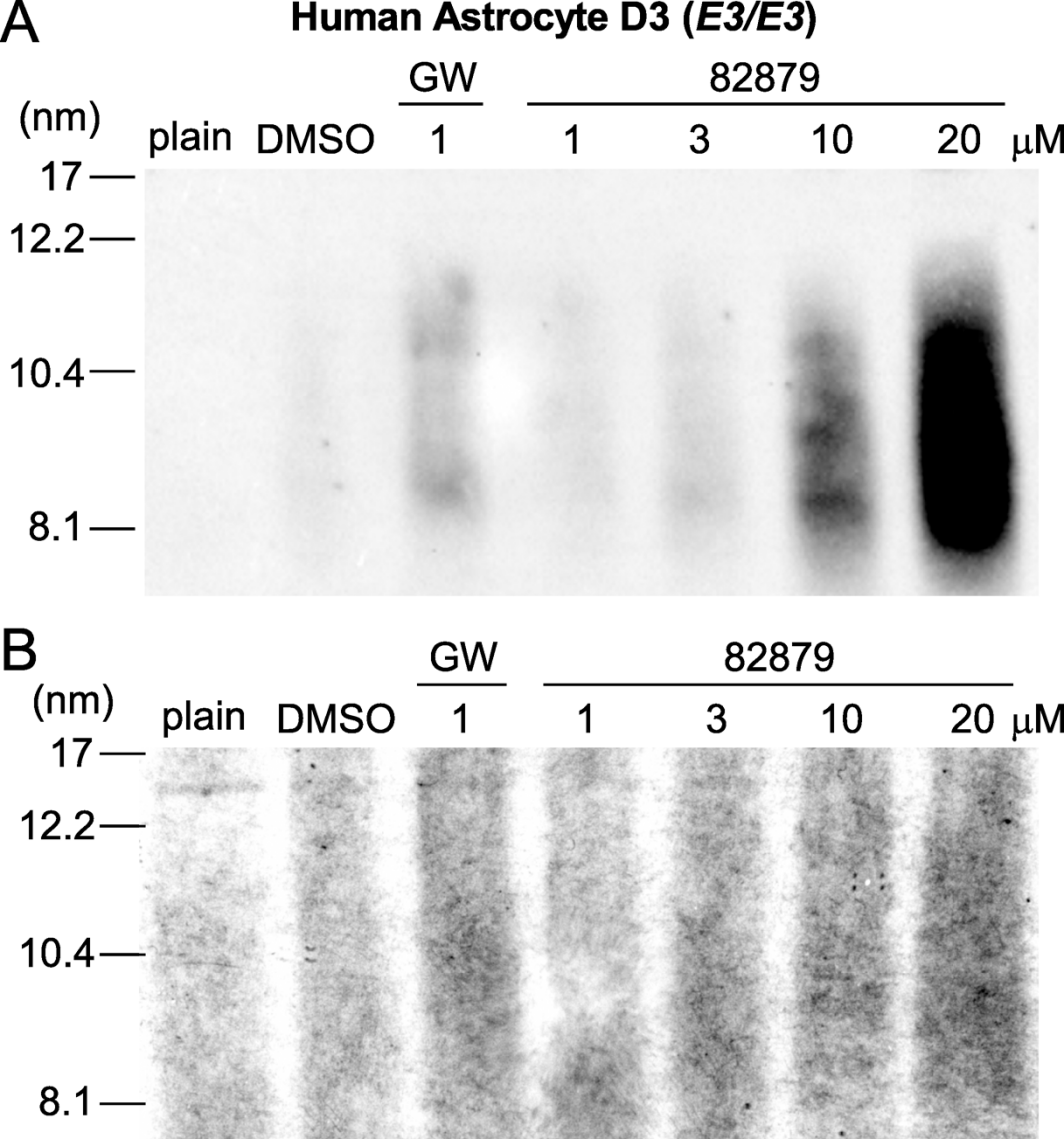
Secreted apoE is found in HDL-like lipoprotein particles following 82879 treatment of primary human astrocytes. Media were collected from human astrocyte donor D3 (APOE3/E3) after 96h treatment with DMSO, GW and 82879 at indicated concentrations. Plain treatment media that was not applied to cells (plain) was also assayed and processed identical to the treated samples. (*A*) The size of apoE-containing lipoproteins was assessed in media samples concentrated 7x and analyzed by 6% native PAGE followed by immunoblotting for apoE. (*B*) Media samples from the same experiment were concentrated 15x and resolved on 6% native PAGE followed by Sudan Black B staining of the gel for total lipids. The Stokes diameter of standards in nm is listed on the left for both figure panels.

To determine whether upregulated total apoE secretion in primary human astrocytes corresponded to increased overall apoE lipidation, as may be expected with elevated ABCA1 levels, and as implied by an increase in both media apoE and media cholesterol in CCF-STTG1 cells, we concentrated media from treated primary human astrocytes and performed non-denaturing gradient gel electrophoresis followed by Western blotting for apoE and Sudan Black B staining for total lipids (Fig 11). Human apoE secreted from murine primary astrocytes is reported to be found in HDL-like sized particles ranging from ~ 8 to 17 nm in diameter [67, 68] similar to what we have observed for mouse apoE secreted from primary murine mixed glia [62]. ApoE within this particle size range increased with higher concentrations of 82879 (Fig 11A). The lipid dye Sudan Black B showed darker staining within this particle size range following both GW3965 treatment and with increasing concentrations of 82879 compared to the background lipid staining in DMSO treated cells and in plain media not exposed to cells (Fig 11B). Our data support that increased apoE and ABCA1 levels correspond to increased secretion of lipidated apoE.

## Discussion

The projected prevalence for AD cases in the aging population coupled with the high failure rate in the AD drug development pipeline has led to an urgent need to identify new AD targets and therapies [3]. ApoE plays an undeniable role in AD pathogenesis and is among the most important targets to understand. Over half of AD patients carry *APOE4*, which confers up to 14.9 times more risk of developing AD and is associated with reduced brain apoE levels and accelerated amyloid deposition [22, 69]. In mouse models of AD-relevant amyloid deposition, direct LXR/RXR agonists increase lipidated apoE, improve memory performance, and can lower amyloid load, suggesting that increasing the levels of functional, lipid-carrying apoE is of therapeutic benefit [70–72]. However, all of the synthetic LXR/RXR agonists used thus far in preclinical studies in AD mice also will inevitably cause hepatotoxicity in humans, an adverse effect that currently precludes further clinical development.

Using a high throughput screen in CCF-STTG1 astrocytoma cells, we identified the chrysanthemic ester 82879 as a compound capable of increasing the levels of secreted lipidated apoE, through mechanisms that likely include both elevated production and lipidation as well as reduced recycling and degradation. CCF-STTG1 cells are reported to be *APOE3/APOE4* [22] and we have independently confirmed this genotype. Riddell and colleagues showed that apoE4 represents <25% of the total apoE secreted from CCF-STTG1 cells and this proportion of secreted apoE4 is retained following treatment with the direct LXR agonist TO901317 [22]. *In vivo*, enhanced intracellular degradation is thought to be the cause of selective reduction in apoE4 levels. Evidence from the human *APOE* targeted replacement EFAD AD mouse model suggests that total brain apoE4 levels are not only lower compared to apoE2 and apoE3, corresponding to higher total Aβ levels in these animals, but that apoE4 may be also be less lipidated [73]. Indeed increasing apoE lipidation, in particular for apoE4, has been proposed as a viable strategy for reducing Aβ accumulation [72]. Notably, compound 82879 also upregulates ABCA1, which is essential for apoE lipidation, thereby having important effects on both apoE stability and function [29–31]. ApoE can also stabilize ABCA1 by decreasing its proteolysis [74]. We confirmed the CCF-STTG1 astrocytoma cell findings in primary human astrocytes, where we observed a robust induction of both apoE and ABCA1. In CCF-STTG1 cells, apoE secretion was associated with increased total media cholesterol levels and, in primary astrocytes, elevated secretion of high molecular-weight apoE corresponded to increased lipid staining in this particle size range. While apoE secretion was increased in both *APOE3* and *APOE4* primary human astrocytes, the single *APOE3/APOE3* donor used in our study had higher basal levels of apoE but showed weaker apoE induction following 82879 treatment.

Although upregulation of ABCA1 by 82879 requires LXRα, induction of apoE expression appears to involve both LXR-dependent and LXR-independent mechanisms. Further studies are necessary to characterize this LXR-independent pathway and determine if it operates in parallel to or intersects with the endogenous LXR signaling axis to modulate apoE expression in astrocytes. That 82879 was the only positive hit validated out of a total of 104,000 compounds in our apoE phenotypic screen and involves LXR signaling for apoE and ABCA1 induction suggests that LXR engagement may be an obligate step for apoE upregulation, particularly important for regulation of lipidated apoE. Indeed our published data [61] and that of other [75] and ongoing apoE phenotypic screens using different libraries support this observation. Importantly, understanding more about the LXR-independent pathways used by 82879 to stimulate apoE expression may yield new targets by which apoE expression and function can be modulated.

Although 82879 shows activity toward LXR target genes in cell types other than astrocytes, importantly, the patterns observed provide support for 82879 having cell-type specific effects that are distinct from GW3965. For example, physiologically relevant primary human macrophages and hepatocytes each showed dose-dependent changes in apoE and ABCA1 mRNA levels upon 82879 treatment. Intriguingly, apoE appears to be a unique target affected by 82879, as apoE mRNA levels showed a dose-dependent increase in human macrophages but a dose-dependent decrease in human hepatocytes, whereas the other LXR targets tested, ABCA1, SREBP-1c and LXRα, all showed the expected increases in expression. As hepatic induction of SREBP-1c is thought to account for much of the undesirable effects of LXR agonists [76], we used both primary human hepatocytes and HepG2 human hepatoma cells in our studies. Unexpectedly, we observed negligible SREBP-1c induction upon 82879 treatment in HepG2 cells. Similarly, little induction of LXR target genes was observed in other immortalized cells including Caco2 and BV2 cells. The mechanisms underlying the distinct responses of these various cell types to 82879 and GW3965 remain to be elucidated, but could include differential drug transporter expression that allow entry of compound into cells, differential metabolism of 82879 and GW3965 in various cell types, and differential expression of transcriptional co-regulators that affect the repertoire of LXR target genes expressed in each cell type.

Compound 82879 is a chrysanthemic ester that shares similarity to the chrysanthemic acid moiety of the pyrethroid structure. Pyrethroid esters are synthetic derivatives of naturally occurring pyrethrins from the chrysanthemum flower [77], which were commercially developed as insecticides with low mammalian toxicity that function by preventing closure of axonal voltage-gated sodium channels in susceptible arthropod species [78, 79]. A defined SAR exists for pyrethroid insecticidal activity, as well as for acute mammalian toxicity [66]. Biological assays comparing 82879 to pyrethroids clearly distinguish two unique aspects of 82879 from pyrethroid ester insecticides in its ability upregulate astrocyte apoE and ABCA1 and its lack of insecticidal activity, whereas the converse biological profile is observed for pyrethroid esters. Notably, as insecticidal activity is mediated though sodium channel and synaptic activity, we predict that 82879 will be negative for these effects. Structurally, the saturated cyclohexyl ring of 82879 is also distinct from other pyrethroid compounds. 82879 has a 1S conformation about the cyclopropane ring that predicts considerably less toxicity than a 1R isomer, as when administered at high doses directly to the CNS, only esters of the 1R cyclopropanecarboxylates are reported to be neurotoxic, whereas the corresponding 1S cyclopropanecarboxylates are without measurable toxicity [66]. In addition to the chyrsanthemic acid moiety, 82879 contains an isoprenoid-like unit found in many natural compounds considered to be health-promoting [80].

Future studies aim to improve potency, initiate pharmacokinetic and pharmacodynamic studies in preparation for in vivo evaluation, and understand the mechanisms of action of compound 82879 to delineate additional novel upstream pathways that regulate apoE in human glia. It will also be important to evaluate functional outcomes following apoE upregulation and determine whether they are affected by an apoE isoform. Our work defines compound 82879 as a new research tool to modulate apoE expression in astrocytes and represents a promising first step towards discovering new AD therapies that target the apoE pathway.

## Supporting Information

S1 Dataset Minimal data set for this study. (XLSX)

## Abbreviations

AD: Alzheimer’s disease
apoE: apolipoprotein E
LXR: liver x receptor
ABCA1: ATP-binding cassette transporter A1
IDOL: inducible degrader of LDRL
CNS: central nervous system
HDL: high-density lipoprotein
LDLR: low-density lipoprotein receptor
BBB: blood brain barrier
RXR: retinoid x receptor
SREBP-1c: sterol regulatory element binding protein 1c
MEF: mouse embryonic fibroblasts
